# Distribution Patterns of Infection with Multiple Types of Human Papillomaviruses and Their Association with Risk Factors

**DOI:** 10.1371/journal.pone.0014705

**Published:** 2011-02-17

**Authors:** Sara Soto-De Leon, Milena Camargo, Ricardo Sanchez, Marina Munoz, Antonio Perez-Prados, Antonio Purroy, Manuel Elkin Patarroyo, Manuel Alfonso Patarroyo

**Affiliations:** 1 Molecular Biology Department, Fundacion Instituto de Inmunologia de Colombia, Bogota, Cundinamarca, Colombia; 2 School of Medicine and Health Sciences, Universidad del Rosario, Bogota, Cundinamarca, Colombia; 3 School of Medicine, Universidad Nacional de Colombia, Bogota, Cundinamarca, Colombia; 4 Mathematics Department, Universidad Publica de Navarra, Pamplona, Navarra, Spain; Rocky Mountain Laboratories, NIAID, NIH, United States of America

## Abstract

**Background:**

Infection with multiple types of human papillomavirus (HPV) is one of the main risk factors associated with the development of cervical lesions. In this study, cervical samples collected from 1,810 women with diverse sociocultural backgrounds, who attended to their cervical screening program in different geographical regions of Colombia, were examined for the presence of cervical lesions and HPV by Papanicolau testing and DNA PCR detection, respectively.

**Principal Findings:**

The negative binomial distribution model used in this study showed differences between the observed and expected values within some risk factor categories analyzed. Particularly in the case of single infection and coinfection with more than 4 HPV types, observed frequencies were smaller than expected, while the number of women infected with 2 to 4 viral types were higher than expected. Data analysis according to a negative binomial regression showed an increase in the risk of acquiring more HPV types in women who were of indigenous ethnicity (+37.8%), while this risk decreased in women who had given birth more than 4 times (−31.1%), or were of mestizo (−24.6%) or black (−40.9%) ethnicity.

**Conclusions:**

According to a theoretical probability distribution, the observed number of women having either a single infection or more than 4 viral types was smaller than expected, while for those infected with 2–4 HPV types it was larger than expected. Taking into account that this study showed a higher HPV coinfection rate in the indigenous ethnicity, the role of underlying factors should be assessed in detail in future studies.

## Introduction

Cervical cancer (CC) is one of the leading causes of cancer-related death among women in developing countries [1]. Women in South and East Africa, Central America and South America are at greater risk of developing cervical cancer, with an average annual incidence of 40 cases per 100,000 women [2]. In Colombia, this disease has become a serious public health problem, with an annual incidence of nearly 18.2 cases of cervical cancer for every 100,000 women [3].

Persistent infection with one of the fifteen oncogenic types of Human Papillomaviruses (HPVs) (commonly denoted as high-risk HPV types (HR-HPV)) is considered the main risk factor associated with this disease [4], [5]. Different epidemiological studies have suggested that about 50–75% of sexually active women are infected with HPV at some point of their lives [6], [7]. However, distribution and prevalence of HPV infection rates vary largely between geographical regions worldwide, probably as a consequence of the numerous factors associated with HPV infection [8], [9]. Even though more than 180 types of HPV have been described, 15 of them are of higher clinical interest due to their high association with malignant disease and their strong mucosal tropism. Furthermore, the burden of disease is mostly shared by 6 types (HPV-16, -18, -31, -33, -45, -58) which are commonly reported in scientific literature as responsible for the majority of lesions [9].

An important risk factor for developing cervical cancer is coinfection, defined as infection with more than one HPV type. It has been established that there is an important association between the number of viral types at the site of infection and the severity of the cervical intraepithelial neoplasia [10], [11]. Of the total number of women infected with HPV, about 20–50% is believed to be infected with more than one viral type [12]–[14].

Other factors which contribute to the risk of developing cervical cancer have been described, including age [15]; the lifetime number of sexual partners, which has been especially associated with coinfection [10]; cigarette smoking, although data regarding this aspect is controversial [16]; prolonged use of hormonal contraceptives (reviewed in [2], [16]); and a large number of full-term pregnancies [17], among others. Recently, Almonte *et al*. divided risk factors for Latin American populations into two groups. The group of short-term risk factors included those related to sexual behaviors, such as age at first sexual intercourse, lifetime number of sexual partners and the sexual behavior of such partners; while the group of long-term risk factors comprised the use of oral contraceptives, high parity and cigarette smoking as cofactors in cervical cancer etiology [18].

Nonetheless, these data are not definitive since discrepancies concerning the factors that favor an increase in the severity of cervical intraepithelial lesions exist, which are most likely related to differences between the methodologies used for collecting samples and laboratory detection methods [19], as well as between women sociodemographic characteristics and sexual behaviors [20]. The two later discrepancies stress the importance of establishing associations between clinical and sociodemographic data with molecular findings in HPV infection events, specific for genetically diverse populations as the ones found in our country.

**Figure 1:**
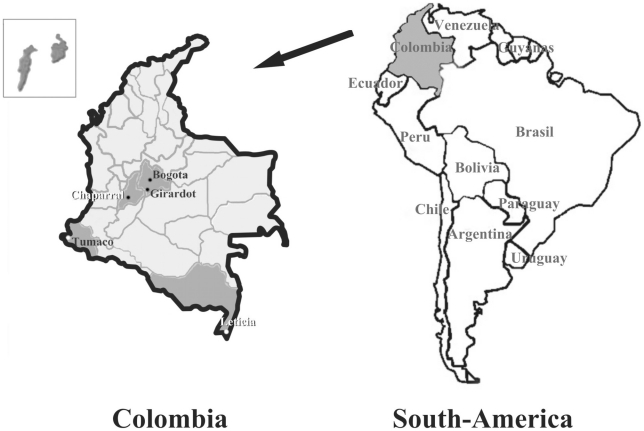
Geographic localization of the five populations included in this study (modified from [49]).

The present study involved 5 regions with different socio-cultural and geographical characteristics, whose CC mortality rates ranged between 3.12 and 5.67/100,000 inhabitants. These regions are: Bogota, the country's capital, with an urban population, which have access to adequate health facilities. Leticia, localized in the department of Amazonas, which is a tropical region inside the colombian jungle characterized by its proximity to Peru and Brazil, favored by high migration rates and an important ethnical diversity with a predominant indigenous background. Chaparral, located in the department of Tolima, is mainly constituted by mestizos with a sedentary life style. Another region participating in this study was Girardot, located in the department of Cundinamarca which due to its favorable weather and great proximity to Bogota has become a preferred touristic destination. It is worth noting that most of the afore-mentioned regions display a predominantly mestizo population, whereas the fifth region considered in this study, Tumaco, which is a Pacific coastal region, represents most of the black population enrolled for this work (Figure 1).

The aim of this study was to assess the number of simultaneous infections in a sample of 1,810 colombian women, which as mentioned in the previous paragraph, come from different regions. With these data, we determined epidemiological profiles of some risk factors associated with possible coinfection patterns.

## Results

Of the total number of women who voluntarily agreed to participate in this study (*n* = 2,110), cervical samples collected from 1,815 women had optimal DNA quality according to β globin gene amplification. Five samples were excluded from the analysis taking into account that they were reported as undetermined in the specific viral type identification; therefore, 1,810 were considered for the statistical analysis. The mean age of these women was 39±12 years, being the youngest 14 years old and the oldest 77 years old. Sociodemographic and clinical data of women participating in this study are shown in Table 1.

**Table 1:** Sociodemographic characteristics, HPV DNA testing and Pap testing results for the 1810 women included in this study.

Characteristics		n (%)*
	<20	57 (3%)
Age	20–40	865 (49%)
	>40	849 (48%)
Smoking	Yes	198 (11%)
	No	1607 (89%)
Lifetime	1	745 (43%)
number of	2–3	816 (47%)
sexual partners	>3	179 (10%)
Age at first	≥18	860 (49%)
intercourse	<18	895 (51%)
	0	116 (7%)
Pregnancies	1–2	619 (36%)
	3–4	618 (35%)
	>4	388 (22%)
	White	302 (17%)
Ethnicity	Indigenous	46 (3%)
	Mestizo	1054 (59%)
	Black	378 (21%)
	None	728 (42%)
Contraceptive	Hormonal	185 (11%)
method	Surgery	536 (31%)
	Barrier	277 (16%)
	Single	299 (17%)
	Married	408 (23%)
Marital status	Civil Union	896 (50%)
	Divorced	137 (8%)
	Widow	45 (2%)
	Normal Cytology	1564 (91%)
Pap smear	ASCUS**	92 (5%)
results	LSIL†	49 (3%)
	HSIL‡	13 (1%)
	HPV-16	834 (42%)
Typing results	HPV-18	268 (14%)
for HPV-DNA	HPV-31	290 (15%)
positive	HPV-33	194 (10%)
samples§	HPV-45	172 (9%)
	HPV-58	94 (5%)
	HPV-6/11	100 (5%)
TOTAL		1810

*Categories have a size lower than 1810, given that there are missing data on the surveys.

**Atypical squamous cells of undetermined significance (ASCUS).

† Low squamous intraepithelial lesion (LSIL).

‡ High squamous intraepithelial lesion (HSIL).

§ Percentages correspond to infection events with each HPV type, calculated over a total of 1952 infections, given that some women were coinfected.

The HPV prevalence among these women was 49.9% (CI 95%: 47.1%–51.7%), with HPV-16 being the most frequently detected type (Table 1). Of the total number of infected women, 65.2% (CI 95%: 62%–68.3%) were infected with multiple HPV types. A previous study on this population had found that infections with multiple HR-HPV types are more frequently associated with types belonging to the A7 species [14]. Compared to expected frequencies and assuming a Negative Binomial distribution, the observed number of women having a single infection (one HPV type) was significantly smaller than expected. For infections with two, three or four HPV types, observed frequencies were generally larger, while detection of more than four viral types in a single woman was much lower than expected (Table 2).

**Table 2:** Number of Observed and Expected data according to a Negative Binomial distribution.

Negative Binomial distribution frequencies		Number of viral types detected by PCR in the cervical sample													
		0		1		2		3		4		>4		χ2	
CHARACTERISTICS		Obs	Esp	Obs	Esp	Obs	Esp	Obs	Esp	Obs	Esp	Obs	Esp	(5df)	p =
	**<20**	27	26	8	14	9	8	10	4	3	2	0	1	10.74	p = 0.05
**Age**	**20–40**	434	430	161	216	150	109	73	55	40	27	7	14	12.94	**p = 0.02**
	**>40**	436	405	133	212	132	111	77	58	53	30	18	16	11.17	p = 0.05
**Smoking**	**Yes**	105	100	33	49	33	25	15	12	9	6	3	3	12.94	**p = 0.02**
	**No**	808	779	278	401	264	207	147	107	88	55	22	28	12.5	**p = 0.03**
**N° Lifetime**	**1**	362	346	115	185	128	99	83	53	48	28	9	15	11.09	p = 0.05
**sexual**	**2–3**	439	417	145	204	122	100	62	49	34	24	14	12	21.32	**p = 0.01**
**partners**	**>3**	88	87	35	45	28	23	15	12	12	6	1	3	12.5	**p = 0.03**
**Age at first**	**≥18 years**	441	431	158	215	139	107	76	54	36	27	10	13	12.94	**p = 0.02**
**intercourse**	**<18 years**	452	424	140	223	145	117	84	62	60	32	14	17	11.12	p = 0.05
	**0**	48	51	25	29	22	16	9	9	9	5	3	3	10.78	p = 0.06
**Pregnancies**	**1–2**	316	306	104	155	104	78	63	40	26	20	6	10	12.55	**p = 0.03**
	**3–4**	309	298	113	154	94	80	55	41	38	21	9	11	11.17	p = 0.05
	**>4**	208	191	59	97	59	49	33	25	22	13	7	6	12.55	**p = 0.03**
	**White**	150	131	36	74	41	42	36	24	28	13	11	8	7.2	p = 0.20
	**Indigenous**	17	17	4	11	8	7	9	4	6	3	2	2	4.75	p = 0.44
**Ethnicity**	**Mestizo**	539	524	186	263	177	132	92	67	51	33	9	17	12.94	**p = 0.02**
	**Black**	196	202	82	94	67	44	21	20	9	10	3	4	21.92	**p = 0.01**
	**None**	372	357	128	182	119	93	58	47	40	24	11	12	12.55	**p = 0.03**
**Contraceptive**	**Hormonal**	86	87	34	46	39	24	11	13	13	7	2	4	11.12	p = 0.05
**method**	**Surgery**	282	264	95	134	68	68	52	35	28	18	11	9	12.55	**p = 0.02**
	**Barrier**	136	132	42	69	50	36	34	19	14	10	1	5	11.17	p = 0.05
	**Single**	154	141	43	75	42	39	37	21	18	11	5	6	11.15	**p = 0.01**
**Marital**	**Married**	203	196	65	102	77	53	32	28	28	14	3	7	11.17	p = 0.05
**status**	**Civil Union**	453	447	172	224	151	112	66	56	40	28	14	14	12.94	**p = 0.02**
	**Divorced**	77	71	23	34	15	17	15	8	4	4	3	2	21.32	**p = 0.01**
	**Widow**	19	20	8	11	10	6	5	3	3	2	0	1	10.74	p = 0.05
	**Normal Citology**	787	758	265	391	268	201	136	104	86	54	22	28	11.17	p = 0.05
**Pap smears**	**ASCUS** **	39	44	23	23	16	12	8	7	6	3	0	2	10.88	p = 0.05
**results**	**LSIL** †	22	22	9	12	5	7	9	4	3	2	1	1	8.03	p = 0.16
	**HSIL** ‡	5	5	1	3	1	2	4	2	1	1	1	1	4.28	p = 0.51
**TOTAL**		**916**	**881**	**311**	**452**	**298**	**232**	**162**	**119**	**98**	**61**	**25**	**31**	**12.55**	**p = 0.03**

*Data with p<0.05 are shown in bold types.

**Atypical squamous cells of undetermined significance (ASCUS).

† Low squamous intraepithelial lesion (LSIL).

‡ High squamous intraepithelial lesion (HSIL).

Since the likelihood–ratio test for alpha = 0 (χ^2^(1) = 221.60; *P* = 0.000) indicated over dispersion, a robust negative binomial regression model was applied to predict the number of infections per woman (a count outcome). This model was evaluated considering all explanatory variables (age, smoking, lifetime number of sexual partners, age at first intercourse, number of pregnancies, ethnicity, contraceptive method, marital status and cytology). The regression model that best predicted the number of HPV infecting types included ethnicity and number of pregnancies (Table 3).

**Table 3:** Negative binomial regression model.

X variables	Coef.	P>z	[95% CI]*		%**
1–2 pregnancies	−0.319750	0.004	−0.537142	−0.102358	−27.4
3–4 pregnancies	−0.327596	0.004	−0.553170	−0.102022	−27.9
>4 pregnancies	−0.372914	0.004	−0.629364	−0.116465	−31.1
Indigenous	0.320354	0.041	0.013561	0.627147	37.8
Mestizo	−0.282646	0.001	−0.444652	−0.120641	−24.6
Black	−0.526085	0.000	−0.730887	−0.321284	−40.9

*CI: Confidence Intervals.

**Percent change in expected count for unit increase in X.

The coefficients for this model are shown in Table 3, as the percentage change in the number of HPV infecting types. When all other variables were maintained constant, having had 1 or more pregnancies decreased the expected number of HPV infecting types, compared to women with no pregnancies. Regarding ethnicity, the expected number of HPV infecting types increased in indigenous women by 37.8% when compared to white women, whereas this number decreased in black and mestizo women (40.9% and 24.6%, respectively).

## Discussion

The extended access to HPV vaccines against certain specific HPV types has motivated studies to determine cross-protection conferred by such vaccines and the role of multiple HPV infections in the development of cervical lesions. It has been demonstrated that there is a direct association between the severity of a lesion and coinfection events, which has been shown to depend on the number of HPV infecting types. Such association has been observed both in coinfections with HR-HPV types, as well as with low risk (LR) types [19]. Follow-up studies have reported that those women infected with one HPV type at the beginning of the study have an increased risk of acquiring a second HPV type [21].

Even though it is wide known that HPV infection prevalence varies in different regions given the population characteristics and the techniques used for detection, this study shows similar data to those reported for Latin America in the last two years, in which infection rates equal or greater than 50.0% were found [22], [23]. As has been previously described, using more than one generic primer set allowed us to detect a greater number of infected women [24], as well as a better coinfection estimate [25].

The clinical importance of coinfection in cervical cancer etiology has been cause of extensive debate. There are reports of patients with multiple cervical intraepithelial lesions of different grades [20], but their association with coinfection events is still not clear. Despite coinfection is seldom reported in cervical cancer [26]–[28], several studies have shown an association between HPV coinfection and the increase in the lesion severity [29], [30]. Although the findings mentioned above might seem contradictory, a possible explanation might be that HPV coinfection could favor the settlement of one of the infecting viruses, given that tumors are usually clonal and thus reflect only 1 HPV type. This notion is supported by previous studies where infections caused by some viral types are favored by a previous infection with other types [31]. These data, plus the partial protection of the currently available vaccines, support studying multiple HPV infections more in depth, as well as determine the risk factors associated to them.

A similar HPV prevalence for each Pap smear category was found. The absence of a greater viral presence in the HSIL group might be explained by GP5+/6+ and MY09/11 primer sets' amplification targets, both of which lie within the L1 encoding gene. It has been shown that viral DNA is mainly integrated to the host genome in high-degree lesions, leading to the partial loss of the L1 ORF [32], [33].

In this study, observed frequencies for the number of infecting genotypes according to a binomial negative distribution, were larger than expected for the group of women coinfected with 4 viral types or less, but smaller for the group of women with a single infection; this trend could be considered as another marker for the high frequency of coinfection present in the population studied. In contrast, the group with women coinfected with more than 4 viral types showed observed frequencies lower than expected, which could be attributed to a greater exposure of this population to the most widely distributed viral types.

A previous study reported by Chaturvedi *et al*., has shown a similar HPV distribution pattern between HIV(+) and HIV(-) women [34], leading us to suggest that such pattern is not necessarily related to the host immunological condition, but rather to the natural history of HPV infection; however, more studies including statistical distributions are needed to shed some light on this matter.

An important conclusion obtained from the multivariate analysis was the decreased risk of acquiring more viral types in women who had given birth one or more times. High parity has been usually associated with the risk of developing cervical cancer, based on the notion that a large number of births increases the number of lesions in the birth canal or causes immunosuppression in such area (reviewed in [2]). However, according to a study conducted by Molano *et al*., the risk of acquiring infections with high risk HPV types, as well as coinfections, tends to diminish with the number of births [13]. This agrees with the results of the present study, which show that the risk of being infected with a large number of HPV types decreased to 31.1% in women who had given birth 4 or more times, compared to women who had not given birth. An explanation to this observation could be associated with riskier sexual behaviors in the latter group of women (e.g. large number of sexual partners during a woman's lifetime). However, this conclusion should be taken with caution because there are not sufficient studies relating the number of births with coinfection, or coinfection with hormonal conditions after labor.

Another important aspect to consider is the increase of a 37.8% in the number of infecting HPV types found in women of indigenous ethnicity, and the decrease of 24.6% and 40.9% in women of mestizo and black ethnicities, respectively, compared to white women. These findings could be influenced by sociocultural and demographic conditions intrinsic to these ethnic groups [35]. The indigenous ethnicity appears to play an important role in the association of infection, possibly due to cultural characteristics, such as limited access to prevention and promotion programs of cervical cancer and sexually transmitted diseases, early start of sexual relationships and childbirth, or intrinsic biological characteristics of this particular ethnia, which contribute to the immune system modulation and might suppress the immune response, thus facilitating the viral infection [36], [37].

Regarding this later aspect, few studies have focused on the susceptibility to coinfection in different ethnic groups. There are reports showing that black women display a certain degree of protection against HPV infection when compared to hispanic ones [38], [39], agreeing with our findings. However, other studies carried out with black women reported that infections found in this population are higher [40]. There are no studies which assess the true HPV prevalence in black women in different geographical regions, since most of recent studies carried out with black women are focused in vaccination and post-vaccination monitoring.

Taking into account a study carried out by Kenney in 1996, in which risk factors associated to infections were assessed in different ethnic groups, it can be inferred that black women have more risk to acquire infections due to their sexual behavior. In average, black women have more life time sexual partners than hispanic women, their actual sexual partners have had more lifetime sexual partners and cigarette smoking is higher in this population. On the other hand, black women that acquire infection have been taking oral contraceptives longer than hispanic women who also develop infection [36]. It is necessary to corroborate these statements in the light of statistical significance. HPV infection susceptibility in black women has been also contradictory from the host genetic background, since there are studies that reported HLA-class-II DQB1*03 alleles both as a risk factor [41], [42] and a protective factor [43]. Further studies are required to establish the real role of infection in this ethnic group.

For Latin America in particular, some studies have examined the role of genes, especially HLA haplotypes in the risk of acquiring HPV infections [44]–[46]. Studies conducted in Brazil have reported major histocompatibility complex alleles involved in susceptibility and protection against HPV infections [47]. In Colombia, no studies have been conducted to identify autochthonous genetic factors related to the predisposition to acquire single or multiple HPV infections to date.

We found a strong association between multiple HPV infections and indigenous ethnicity in a previous study, where the same population described here was analyzed [14]; however, no association with other ethnicities was found. The different outcomes might have been due to a different dependant variable, which in this case is the number of HPV infecting types. Low-risk infections were not considered in the previous study.

This study reports an important association between the risk of acquiring infection with multiple HPV types and the indigenous ethnicity, which highlights the importance of focusing studies in this vulnerable population. Nevertheless the transversal design of this study prevents detecting neither acquisition patterns of more than one HPV type nor its relationship with the different risk factors, which make it relevant to conduct longitudinal studies to detect the moment when the acquisition of other HPV types occur and determine if infection events take place simultaneously. Here we report that the infection cases observed with 2 to 4 HPV types are greater than the expected, which suggests the importance of carrying out an analysis of infecting species. Even though we did not assess the distribution of coincident HPV types in our study, our group has reported two studies in which this topic is discussed [14], [48].

The analysis carried out in this study is important since few studies have compared the risk factors associated with single and multiple HPV infections in socioculturally diverse populations as the one existing in Colombia.

## Materials and Methods

### Study population and ethical considerations

This study enrolled women living in five geographical regions of Colombia attending to their cervical screening consult between April and September of 2007. These five populations have been described elsewhere [14]. Prior to undergoing gynecological examination, all women signed a written informed consent and filled out a questionnaire regarding sociodemographic characteristics, sexual behaviors and risk factor data (Table 1). An informed consent was signed by a parent or guardian in the case of children under age. Each women voluntarily agreed to provide a sample of cervical epithelium which was analyzed by Papanicolau testing and HPV–DNA detection. This study was conducted with the supervision and approval of each institution's Ethics Committee, as follows: the Ethics Committee of Fundación Instituto de Inmunología de Colombia, Bioethics Committee of the League Against Cancer - Amazon Sectional, The Ethics Committee of Nuevo Hospital San Rafael E.S.E - Girardot, Bioethics Committee of Hospital San Juan Bautista de Chaparral E.S.E, Hospitalary Ethics Committee of Hospital de Engativá Nivel II and Ethics Committee of Hospital San Andres, Tumaco E.S.E.

### Detection of HPV DNA by PCR amplification

Cervical samples were collected with a cytobrush and stored in 95% ethanol at 4°C [49]. DNA from these samples was digested with lysis buffer containing 10 mM Tris–HCl (pH 7.9), 0.45% Nonidet–P-40, 0.45% Tween 20 and 60 µg/mL of Proteinase K (Invitrogen, California, USA), first at 60°C for 1 h and then at 95°C for 10 min [50]. A 2.7-µL aliquot of each processed sample was amplified by PCR using the human β-globin GH20/PC04 primers to check DNA integrity [51] and two HPV generic primer sets were employed for detection, to prevent underestimation in the determination of viral prevalence caused by the use of a single HPV identification set [52]. These sets are: GP5+/GP6+ which allows detecting low viral loads [51], [53] and MY09/MY11 which has higher sensitivity for detecting infections with more than one viral type [25]. Additionally, these generic primer sets are easily implemented and increase robustness and sensibility to epidemiological studies [24], [54]. Despite the advantages obtained, it is worth noting that the use of multiple primer sets can also increase contamination, which is why protocols aimed at minimizing this contamination must be followed. In the present study, negative and positive controls were run every 46 samples, and whenever either control gave an unexpected result, the whole series was repeated. Samples testing positive with one or both generic primer sets underwent PCR amplification with type-specific primers for HPV-6/-11, -16, -18, -31, -33, -45 and -58 annealing within the E5, E6 and E7 regions [4], [24], [55], [56], Studies carried out in Latin America have found that the 6 high-risk viral types included in this study are present in the 90% of cases of cervical cancer [57]. Synthetic genes encoding HPV-18, -31, -45 and -58 early regions and HPV-6/-11, -16 and -33 infected samples were used as positive controls for type-specific PCR reactions [58]. This PCR procedure was carried out in duplicate and DNA-free water was used as negative control to rule out DNA contamination.

### Statistical analysis

Descriptive statistics were used to evaluate demographic and clinical characteristics of the population. The expected number of coinfecting genotypes was estimated assuming a negative binomial distribution. Although previous studies used a Poisson model to evaluate the fit of data [34], in our sample the over dispersion coefficient alpha was significantly different from zero for all of the variables included in the analysis. The difference between observed vs. expected frequencies was evaluated using a one-way χ^2^ Goodness-of-fit test.

In order to evaluate which explanatory variables (age, smoking, lifetime number of sexual partners, age at first intercourse, number of pregnancies, ethnicity, contraceptive method, marital status and cytology) were associated with the number of coinfections (dependent variable), a negative binomial model was used instead of a Poisson distribution, since this model does not consider the existence of over dispersion (in our data the mean/variance ratio was 0.7) [59]. Additionally, a likelihood ratio test was used to compare the relative fit of the Poisson model to the negative binomial model. The negative binomial model assumes that the number of coinfections is a count outcome. The use of this model is preferred over other tests that estimate ORs, given both the count outcome and the high frequencies of the expected outcomes [60]. All the statistical analyses were performed using STATA 9®.
